# Supplementary material for: Associations of smoking status and leisure-time physical activity with waist circumference change—10-year follow-up among twin adults
Source: Int J Obes (Lond). 2025 Jun 29;49(9):1770–6. doi: 10.1038/s41366-025-01820-7 (PMC12463656; doi:10.1038/s41366-025-01820-7)
Supplement: Supplementary file 1 — Supplement tables [file 41366_2025_1820_MOESM1_ESM.docx]

**Supplement Table 1.** Baseline distributions of covariates among individuals (n=3,322) by their long-term smoking status.

| **Long-term smoking status** | **Persistent smokers ^a^**  **n=643 (19.3%)** | **Quitters ^b^**  **n=273 (8.2%)** | **Non-current smokers ^c^**  **n=1,856 (55.9%)** | **Others ^d^**  **n=550 (16.6%)** |
| --- | --- | --- | --- | --- |
| **Categorical variables** | **n (%)** | **n (%)** | **n (%)** | **n (%)** |
| **Sex** |  |  |  |  |
| Men (n=1,527) | 333 (51.8) | 141 (51.6) | 781 (42.1) | 272 (49.5) |
| Women (n=1,795) | 310 (48.2) | 132 (48.4) | 1,075 (57.9) | 278 (50.5) |
| **Socio-economic status** |  |  |  |  |
| Very good/fairly good | 180 (28.0) | 96 (35.2) | 665 (35.8) | 186 (33.8) |
| Average | 278 (43.2) | 120 (44.0) | 812 (43.8) | 229 (41.6) |
| Fairly bad/very bad | 185 (28.8) | 57 (20.9) | 379 (20.4) | 135 (24.5) |
| **Self-rated health** |  |  |  |  |
| Excellent /very good | 488 (75.9) | 224 (82.1) | 1,634 (88.0) | 477 (86.7) |
| Good | 136 (21.2) | 46 (16.8) | 190 (10.2) | 65 (11.8%) |
| Rather poor/very poor | 19 (3.0) | 3 (1.1) | 32 (1.7) | 8 (1.5) |
| **Sleep problems** |  |  |  |  |
| Not at all/seldom | 441 (68.6) | 201 (73.6) | 1,405 (75.7) | 414 (75.3) |
| Once a week/more often | 202 (31.4) | 72 (26.4) | 451 (24.3) | 136 (24.7) |
| **Continuous variables** | **mean (SD)** | **mean (SD)** | **mean (SD)** | **mean (SD)** |
| **Age** | 24.5 (0.9) | 24.5 (1.0) | 24.4 (0.9) | 24.4 (0.9) |
| **Alcohol use** (g/week) | 96.1 (121.6) | 78.0 (88.5) | 43.1 (60.3) | 68.4 (87.1) |
| **Diet quality** (DQS, sum score) | 7.0 (2.1) | 7.7 (2.1) | 8.2 (2.1) | 7.7 (2.2) |
| **Psychological distress** (GHQ12, sum score) | 11.3 (5.5) | 11.0 (5.7) | 10.4 (5.2) | 10.3 (4.7) |
| **Life satisfaction** (sum score) | 8.9 (3.2) | 9.0 (3.4) | 8.3 (2.9) | 8.3 (3.0) |

^a^ Persistent daily or occasional smoking; ^b^ Quitting from daily smoking; ^c^ Quitting from occasional smoking, consistent former smoking, never smoking; ^d^ Miscellaneous smoking status (initiators, reducers, relapses, increases and other changes in smoking status during the follow-up).

DQS=Diet quality score; GHQ12= General Health Questionnaire 12.

**Supplement Table 2.** Means (SD) of anthropometric measures and leisure-time physical activity at baseline and follow-up by smoking status among individuals (n=3,322).

| **Long-term smoking status** | **Persistent smokers ^a^**  n=643 | **Quitters ^b^**  n=273 | **Non-current smokers ^c^**  n=1,856 | **Others ^d^**  n=550 |
| --- | --- | --- | --- | --- |
| **Measure** |  |  |  |  |
| Height (cm) baseline | 172.4 (9.0) | 172.4 (9.3) | 171.9 (9.2) | 172.9 (9.1) |
| Height (cm) follow-up | 172.4 (9.1) | 172.4 (9.2) | 171.9 (9.2) | 172.9 (9.2) |
| Weight (kg) baseline | 69.2 (13.7) | 68.1 (12.7) | 67.8 (13.4) | 68.7 (13.9) |
| Weight (kg) follow-up | 74.7 (15.5) | 75.4 (15.5) | 72.9 (15.6) | 74.2 (15.9) |
| BMI (kg/m^2^) baseline | 23.1 (3.5) | 22.8 (3.2) | 22.8 (3.3) | 22.8 (3.3) |
| BMI (kg/m^2^) follow-up | 25.0 (4.1) | 25.3 (4.1) | 24.5 (4.3) | 24.7 (4.3) |
| WC (cm) baseline | 80.4 (10.9) | 79.4 (10.0) | 78.9 (10.3) | 79.5 (10.3) |
| WC (cm) follow-up | 87.0 (13.1) | 87.8 (12.3) | 84.9 (12.2) | 86.4 (12.8) |
| ∆ WC (cm) | 6.6 (8.3) | 8.4 (8.1) | 6.1 (7.9) | 6.9 (8.4) |
| LTPA (MET-h/wk) baseline | 22.0 (28.9) | 20.4 (24.2) | 34.3 (35.5) | 31.0 (33.5) |
| LTPA (MET-h/wk) follow-up | 19.3 (25.3) | 25.4 (28.4) | 27.5 (29.1) | 24.7 (27.6) |
| ∆ LTPA (MET-h/wk) | -3.2 (31.8) | 5.0 (35.0) | -8.2 (35.5) | -6.0 (35.3) |

^a^ Persistent daily or occasional smoking.

^b^ Quitting from daily smoking.

^c^ Quitting from occasional smoking, consistent former smoking, never smoking.
^d^ Miscellaneous smoking status (initiators, reducers, relapses, increases and other changes in smoking status during the follow-up).

**Supplement Table 3.** Distribution of numbers of all 1,050 pairs and of 390 monozygotic pairs by their pairwise smoking status. The smoking status of the first twin within a pair is represented by the table rows and the smoking status of the second twin within a pair by table columns. Concordant pairs are on the diagonal and discordant pairs are off diagonal.

| **Smoking status** | **Persistent** | **Quitters** | **Non-current** | **Other** |
| --- | --- | --- | --- | --- |
|  |  |  |  |  |
| **All pairs** |  |  |  |  |
| Persistent smokers | 76 | 45 | 129 | 66 |
| Quitters |  | 16 | 59 | 23 |
| Non-current smokers |  |  | 417 | 178 |
| Other |  |  |  | 41 |
|  |  |  |  |  |
| **Monozygotic pairs** |  |  |  |  |
| Persistent smokers | 40 | 11 | 31 | 31 |
| Quitters |  | 6 | 19 | 7 |
| Non-current smokers |  |  | 166 | 60 |
| Other |  |  |  | 19 |
|  |  |  |  |  |
