# Supplementary material for: Associations of smoking status and leisure-time physical activity with waist circumference change—10-year follow-up among twin adults
Source: Int J Obes (Lond). 2025 Jun 29;49(9):1770–6. doi: 10.1038/s41366-025-01820-7 (PMC12463656; doi:10.1038/s41366-025-01820-7)
Supplement: Supplementary file 2 — Supplement figures [file 41366_2025_1820_MOESM2_ESM.docx]

**PICTORIAL INSTRUCTION FOR WAIST CIRCUMFERENCE MEASUREMENT**

Finally, we ask you to measure round your waist with the measuring tape we have sent you earlier along with the invitation letter. Please stand up straight when measuring. Measure the slimmest point of your waist. If you have difficulties in finding it, measure as shown at the picture, the circumference situated in the middle of the lowest part of the ribs (A) and the upper part of the hip bone (B).

My waist measurement is _______ cm


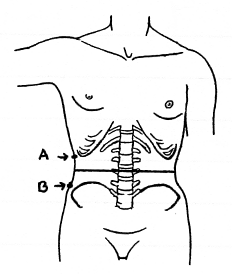


**Supplement Figure 1.** Pictorial instruction for waist circumference self-measurement.

**Supplement Figure 2**. Box-plots with the median for change in waist circumference (WC) with 75^th^ percentiles by smoking status during 10-year follow-up.

**Supplement Figure 3.** Change in waist circumference (WC) among 45 discordant twin pairs (persistent smokers *versus* quitters).

MZ = monozygotic (n=11 pairs); ALL DZ = All dizygotic (n=34 pairs); SSDZ=same-sex dizygotic (n=22 pairs); OSDZ=opposite-sex dizygotic (n=12 pairs)
